# Baclofen-associated neurophysiologic target engagement across species in fragile X syndrome

**DOI:** 10.1186/s11689-022-09455-9

**Published:** 2022-09-27

**Authors:** Carrie R. Jonak, Ernest V. Pedapati, Lauren M. Schmitt, Samantha A. Assad, Manbir S. Sandhu, Lisa DeStefano, Lauren Ethridge, Khaleel A. Razak, John A. Sweeney, Devin K. Binder, Craig A. Erickson

**Affiliations:** 1 Division of Biomedical Sciences, School of Medicine, University of California, Riverside, USA; 2 Division of Child and Adolescent Psychiatry, Cincinnati Children’s Hospital Medical Center, Cincinnati, OH USA; 3 Division of Neurology, Cincinnati Children’s Hospital Medical Center, Cincinnati, OH USA; 4 Department of Psychiatry and Behavioral Neuroscience, University of Cincinnati College of Medicine, Cincinnati, OH USA; 5 Division of Developmental and Behavioral Pediatrics, Cincinnati Children’s Hospital Medical Center, Cincinnati, OH USA; 6 Department of Pediatrics, University of Cincinnati College of Medicine, Cincinnati, OH USA; 7 Department of Psychology, University of Oklahoma, Norman, OK USA; 8 Department of Pediatrics, University of Oklahoma Health Sciences Center, Oklahoma City, OK USA; 9 Neuroscience Graduate Program, University of California, Riverside, USA; 10 Psychology Graduate Program, University of California, Riverside, USA

**Keywords:** Fragile X syndrome, Electroencephalography, Biomarker, Baclofen, Autism, Multielectrode array

## Abstract

**Background:**

Fragile X syndrome (FXS) is the most common inherited form of neurodevelopmental disability. It is often characterized, especially in males, by intellectual disability, anxiety, repetitive behavior, social communication deficits, delayed language development, and abnormal sensory processing. Recently, we identified electroencephalographic (EEG) biomarkers that are conserved between the mouse model of FXS (*Fmr1 KO* mice) and humans with FXS.

**Methods:**

In this report, we evaluate small molecule target engagement utilizing multielectrode array electrophysiology in the *Fmr1 KO* mouse and in humans with FXS. Neurophysiologic target engagement was evaluated using single doses of the GABA_B_ selective agonist racemic baclofen (RBAC).

**Results:**

In *Fmr1* KO mice and in humans with FXS, baclofen use was associated with suppression of elevated gamma power and increase in low-frequency power at rest. In the *Fmr1* KO mice, a baclofen-associated improvement in auditory chirp synchronization was also noted.

**Conclusions:**

Overall, we noted synchronized target engagement of RBAC on resting state electrophysiology, in particular the reduction of aberrant high frequency gamma activity, across species in FXS. This finding holds promise for translational medicine approaches to drug development for FXS, synchronizing treatment study across species using well-established EEG biological markers in this field.

**Trial registration:**

The human experiments are registered under NCT02998151.

**Supplementary Information:**

The online version contains supplementary material available at 10.1186/s11689-022-09455-9.

## Background

Fragile X syndrome (FXS) is the most common inherited genetic cause of intellectual disability and most common single gene cause of autism spectrum disorder (ASD) [1]. FXS is caused by a CGG repeat expansion and subsequent methylation in the *fragile X mental retardation 1 (Fmr1)* gene that results in deficient production of fragile X protein (FXP; formerly termed fragile X mental retardation protein (FMRP)) [2]. FXP is an RNA-binding protein that regulates synaptic function through regulation of protein translation [3]. In addition to intellectual disability, clinical features associated with FXS often include increased anxiety, repetitive behaviors, social communication deficits, delayed language development, and abnormal sensory processing [4–15]. Using EEG, our group and others have identified abnormal sensory processing in FXS including hypersensitivity and reduced habituation to repeated stimuli [16–18].

Identification of comparable biomarkers in humans and validated animal models is a critical step in facilitating pre-clinical to clinical therapeutic pipelines to advance treatment development for neurodevelopmental disorders, as many novel therapeutics showing promise in rodent models have failed in clinical trials in humans with FXS [15, 19–21]. To provide relevant translational electrophysiological biomarkers, we have developed and applied multielectrode array (MEA) analysis in *Fmr1* KO mice, the mouse model of FXS [22, 23]. Our murine system involves stable chronic in vivo implantation of a planar multielectrode array (MEA) on the surface of the mouse skull and enables low-noise 30-channel simultaneous EEG, which can then be used for acquiring resting and stimulus-evoked EEG in awake, freely moving mice [22]. In humans, we have utilized 128-channel high-density EEG sampling. In mouse and human studies, we use analogous paradigms for auditory stimulation [24], and in both species have demonstrated robust phenotypes of altered resting EEG power, particularly in the gamma frequency band, altered single-trial and train-related EEG power and reduced inter-trial phase coherence (ITPC) to auditory chirp stimuli [23, 24]. Similar resting state and auditory evoked response EEG findings have been noted in FXS across species [18, 23–29], identifying cross-species electrophysiology as a particularly promising translational treatment development biomarker tool in the field.

In this study, we test a specific candidate mechanism for electrophysiological target engagement across mouse and human study in FXS. Deficient GABA neurotransmission has been implicated in the pathophysiology of FXS [30–32]. Pharmacological enhancement of GABAR subtypes shows efficacy against pathological behaviors and brain hyperexcitability in FXS and *Fmr1* KO mice [33–36]. Proof-of-principle for salutary effects of the GABA_B_ agonist racemic baclofen (RBAC) on EEG and behavior in *Fmr1* KO mice was observed previously [36]. In that study, RBAC was found to suppress gamma power and improve working memory and anxiety-related behavior in a dose-dependent manner in *Fmr1* KO mice. Thus, evidence exists that baclofen may be therapeutic in FXS. However, single-dose RBAC has not been tested in parallel in animal models of FXS and patients with FXS. In this study, we administered acute RBAC to *Fmr1* KO mice and to humans with FXS, and examined the effects of the molecule on translational EEG biomarkers. We demonstrate significant dose-related reduction in EEG gamma power and amelioration of inter-trial phase coherence (ITPC) deficits to temporally modulated auditory stimuli by single dose racemic baclofen in *Fmr1* KO mice, and corresponding reduction in gamma power following single-dose RBAC in humans with FXS.

## Methods

### Mouse

**Fig. 1:**
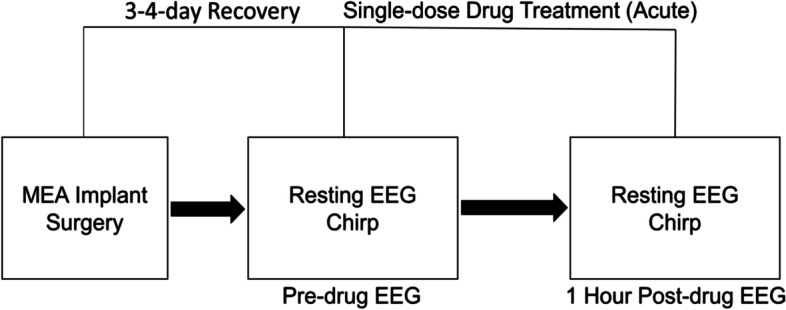
Mouse experimental design. After 4 days of recovery from multi-electrode array (MEA) implantation, EEG responses were recorded and served as pre-drug baseline responses. Post-drug EEG responses were recorded after acute single-dose drug treatment

Male *Fmr1* KO (B6.129P2-Fmr1tm1Cgr/J, stock #003025) [37] and C57BL/6J WT (stock #000664) mice were obtained from The Jackson Laboratory. All genotypes were confirmed by Transnetyx (Cordova, TN) using real-time PCR analysis. Mice were maintained in an AAALAC-accredited facility under a 12-h light/dark cycle and were provided irradiated rodent diet (PicoLab, 5053) and water ad libitum. All mouse procedures were performed with approval from the University of California Institutional Animal Care and Use Committee and in accordance with the NIH Animal Care and Use Guidelines. EEG recordings were obtained from 20 *Fmr1* KO and 20 WT mice. Male mice between 12 and 15 weeks of age were used for all EEG recordings. In each group (*n* = 10 WT, *n* = 10 *Fmr1* KO), EEG data were recorded 3–4 days after recovery from MEA implantation surgery and served as pre-drug baseline responses (“pre-drug EEG”, Fig. 1). EEG recordings were obtained from the same mice one hour after racemic baclofen (RBAC) treatment (2.5 mg/kg or 5 mg/kg, i.p.) (“1 h post-drug EEG”, Fig. 1). The 5 mg/kg dosage is the *Fmr1* KO mouse approximate equivalent to our 30 mg dosing in humans with FXS [38]. Racemic baclofen (Sigma #B5399-5G) solutions were suspended using saline vehicle. EEG recordings obtained during each mouse recording session included resting-state EEG and auditory chirp stimuli (see below). Recordings were collected using the SmartBox (Neuronexus) acquisition system from awake and freely moving mice [22, 23]. Acquisition hardware was set to lower (0.5 Hz) and upper (500 Hz) filters and data were sampled at a rate of 1250 Hz. MEA surgical and recording procedures followed our previously published methods [39, 40].

### Human

In humans, we analyzed neural correlates of a RBAC acute dose (30 mg) challenge in a single-dose placebo-controlled crossover study with a 2-week washout period. For the human study, legally authorized representatives and participants provided informed consent/assent for the completion of all study procedures and the human work was reviewed and approved by the Cincinnati Children’s Hospital Institutional Review Board (IRB; study registered at clinicaltrials.gov as NCT02998151). Seventeen adolescents and adults with full mutation FXS received single dose (30 mg; equivalent of 5 mg/kg mouse dose) or placebo in random order (see Table 1 for participant characteristics). Resting-state and auditory chirp paradigms were performed in humans as previously described [18, 25], with presentation and analyses directly paralleling our mouse studies. Recordings were collected with a Phillips/EGI NetAmp 400 system (Eugene, Oregon, USA) using a 128-channel Hydrocel saline-based electrode net sampled at 1000 Hz.

**Table 1:** Human subject characteristics

**Mean (SD)**	**FXS (*n*= 17)**
**Age**	**26.3 (8.9)** **Range 16–43**
**% male (*n*)**	**69 (11)**
**Stanford-Binet 5 Abbreviated IQ (SS)**	**57.5 (17.0)** **Range 47–88**
**Stanford-Binet 5 Deviation IQ**	**55.9 (29.5)** **Range 11–91**

### Resting-state

Mouse resting EEG data was analyzed for 2 factors: treatment (pre, post) and frequency (delta to gamma) for the cortical regions (left frontal, right frontal, left medial, right medial, left temporal and right temporal). Data were expressed as the ratio of pre-treatment (Pre) values to gauge relative differences in various factors using the same scale. Mouse Pre data for each frequency band were normalized to 1. Mouse EEG data analysis was performed using a combination of Analyzer 2.1 (Brain Vision Inc.), MATLAB, and SPSS. Data were extracted from the Smartbox files and saved in a file format compatible with Analyzer 2.1 software. Data were first down sampled to 625 Hz and a 60 Hz notch filter was used. EEG artifacts were removed using a semi-automatic procedure in Analyzer 2.1 for all recordings. Less than 20% of data were rejected due to artifacts from any single mouse. Resting-state (no auditory stimulus) EEG data were divided into 1 s segments and fast Fourier transforms (FFT) was run on each segment using a 10% Hanning window at 0.5 Hz bins resolution and then average power (μV/Hz^2^) was calculated for each mouse from 1 to 100 Hz. Power was then further binned into standard frequency bands: Delta (1–4 Hz), Theta (4–8 Hz), Alpha (8–13 Hz), Beta (13–30 Hz), Low Gamma (30–55 Hz), and High Gamma (65–100 Hz). We analyzed the raw data using two-way ANOVA.

In humans, for resting-state EEG analysis current source density (CSD) was estimated from eighty seconds of continuous preprocessed EEG data using a minimum norm estimate (MNE) model. Surface was parcellated into cortical nodes and grouped into bilateral regions (frontal, parietal, temporal, and occipital) according to the Desikan-Killiany atlas [41]. Relative power (band specific power divided by total power) was calculated for each frequency band and a linear mixed effect model (LMM) was performed to account for individual differences between participants using an alpha level of .05 and a false discovery rate (FDR) of 5%. The LMM examined fixed effects of changes in power (post-dose–pre-dose) across 2 conditions (placebo or RBAC), 6 frequency bands (akin to the mouse work above: Delta (1–4 Hz), Theta (4–8 Hz), Alpha1 (7.5–10), Alpha2 (10–13 Hz), Beta (13–30 Hz), Low Gamma (30–55 Hz), and High Gamma (65–100 Hz)) across 8 bilateral cortical regions with nodes serving as replicates and subject as a random effect. To visualize the results, we implemented the R function *scale* to numerically scaled and centered change values for each subject by frequency band and divided it by the max value to ensure boundaries of − 1 to 1.

### Chirp

Across species, following 5 min of resting-state recording we used a chirp-modulated tone (henceforth, ‘chirp’) to induce synchronized oscillations in EEG recordings [24]. The chirp stimulus used was broadband noise whose amplitude was modulated by a sinusoid with linearly increasing frequencies from 1 to 100 Hz [42–44]. Each stimulus was 2 s in duration, and the depth of modulation was 100%. For mice, chirp trains were presented via speaker positioned at the floor of the recording chamber at ~ 70 dB SPL 300 times with the interval between each train randomly generated to be between 1 and 1.5 s. We confirmed in each case that this dB level did not induce audiogenic seizures. For humans, chirp trains were presented via headphones at 65 db SPL 200 times each with the interval between each train randomly generated to be between 1.5 and 2 s.

The chirp facilitates a rapid measurement of transient oscillatory entrainment (delta to gamma frequency range) to auditory stimuli of a wide range of frequencies and can be used to compare oscillatory responses in different groups in clinical and pre-clinical settings [44]. Inter-trial phase coherence analysis (phase locking factor) [45] can then be used to determine the ability of neural generators to synchronize oscillations to the frequencies present in the auditory stimulus.

Across species, chirp trains were processed with Morlet wavelets linearly spaced from 1 to 100 Hz using voltage (μV) and wavelet coefficients were exported as complex values for use with Inter-trial phase coherence (ITPC) analysis. Wavelets were run with a Morlet parameter of 10. This parameter was chosen since studies in humans found most robust difference around 40 Hz, where this parameter is centered [24]. To measure phase synchronization at each frequency across trials, inter-trial phase coherence (ITPC) was calculated. The equation used to calculate ITPC is:

$$ITPC\left(f,t\right)=\frac{1}{n}\sum \limits_{k=l}^n\frac{F_k\left(f,t\right)}{\left|{F}_k\left(f,t\right)\right|}$$

where *f* is the frequency, *t* is the time point, and *k* is trial number. Thus, *F*_*k*_*(f,t)* refers to the complex wavelet coefficient at a given frequency and time for the *k*th trial.

Specifically in mice, there were no less than 275 chirp trials (out of 300) for any given mouse after segments containing artifacts were rejected. For the mice, statistical group comparisons of ITPC in chirp trains were quantified using a Monte Carlo permutation approach. Analysis was conducted by binning time into 256 parts and frequency into 100 parts, resulting in a 100 × 256 matrix. Non-parametric analysis was used to determine contiguous regions in the matrix that were significantly different from a distribution of 2000 randomized Monte Carlo permutations based on previously published methods. Cluster sizes of the real treatment assignments (both positive and negative direction, resulting in a two-tailed alpha of *p* = 0.025) that were larger than 97.25% of the random group assignments, were considered significantly different between experimental conditions. This method avoids statistical assumptions about the data and corrects for multiple comparisons. For human data, theta/alpha ITPC to stimulus onset, ITPC to the chirp stimulus at 40 Hz, ITPC to the chirp stimulus centered at 80 Hz, and single trial power across the entire trial in the alpha and gamma bands were examined in two frontal electrodes that have previously been included in a montage used to examine auditory event related activity (F3, left hemisphere; F4, right hemisphere; see [18] for details). Each human variable was submitted to a 2 (drug vs placebo) × 2 (pre-dose vs post-dose) × 2 (hemisphere) repeated measures ANOVA with an alpha level of .05 (see [24] for additional detail). All 17 participants with FXS provided chirp data for at least 3 out of four sessions, however only 11 participants provided a complete chirp dataset with at least 35% artifact-free trials at every session, therefore only 11 participants are included in the human chirp statistical analyses.

## Results

### EEG response to acute RBAC treatment in mice

#### Effects of 2.5 mg/kg (low dose) racemic baclofen on EEG in WT and KO mice

**Fig. 2:**
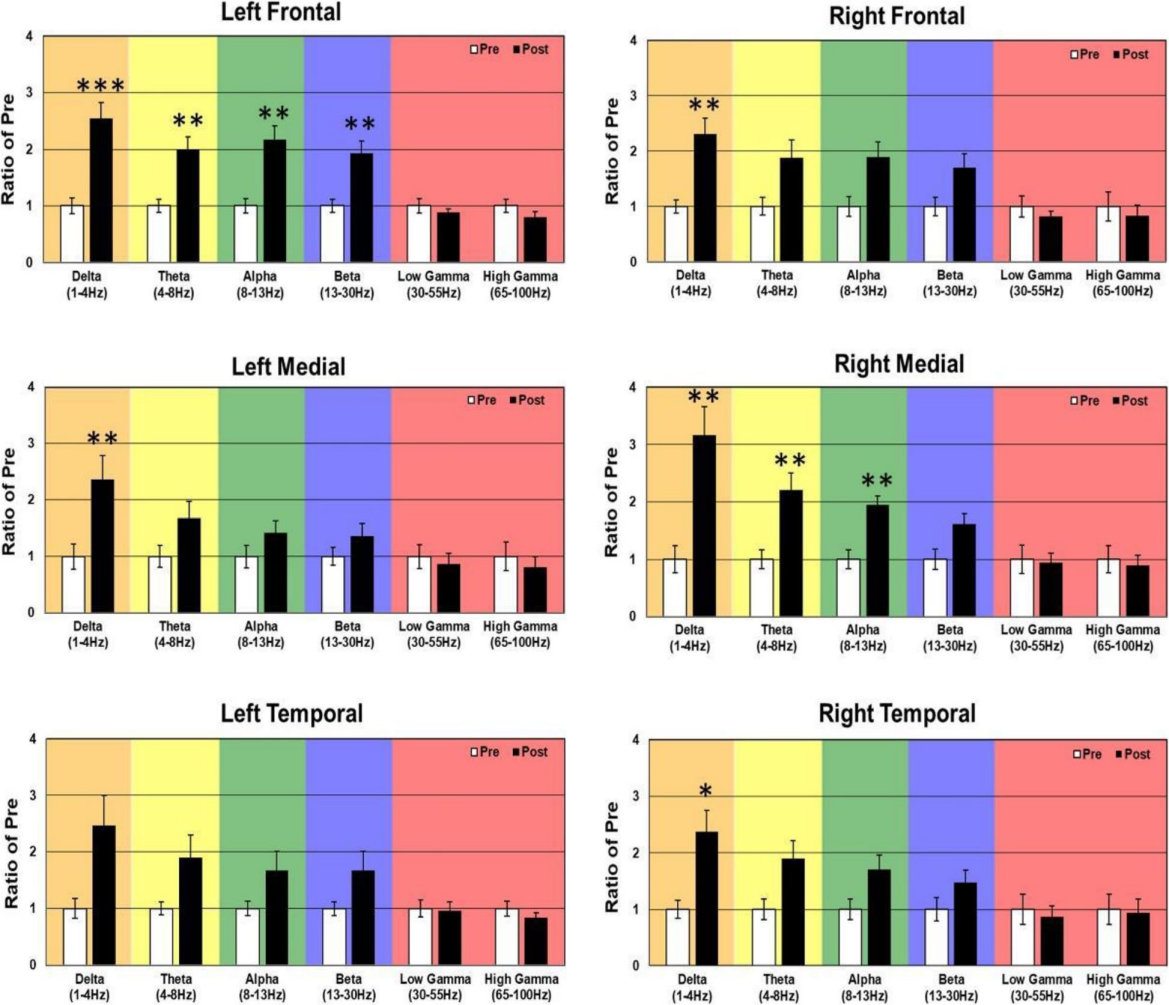
Effect of 2.5 mg/kg on EEG in WT mice. Ratio of WT post 2.5 mg/kg low dose RBAC to pre-EEG power across frequency bands for distinct cortical regions following treatment

**Fig. 3:**
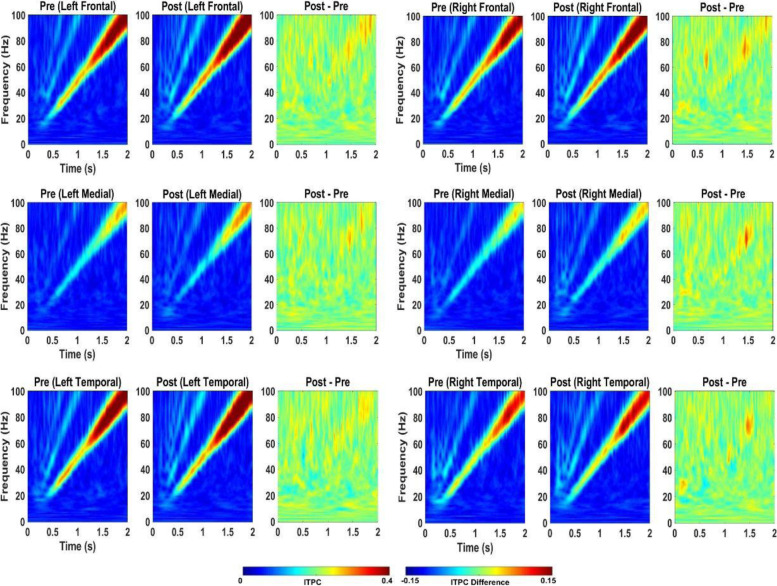
Effect of 2.5 mg/kg RBAC on auditory chirp in WT mice. For each cortical region, the left panel shows the averaged inter-trial phase coherence (ITPC or phase locking factor) before vehicle treatment (Pre), the middle panel shows the averaged ITPC after vehicle treatment (Post) and the right panel shows Post–Pre. Significant increases in ITPC in Post compared to Pre are shown in black-outlined areas. Red areas in the right panels (Post–Pre) represent positive ITPC differences and blue areas represent negative ITPC differences

**Fig. 4:**
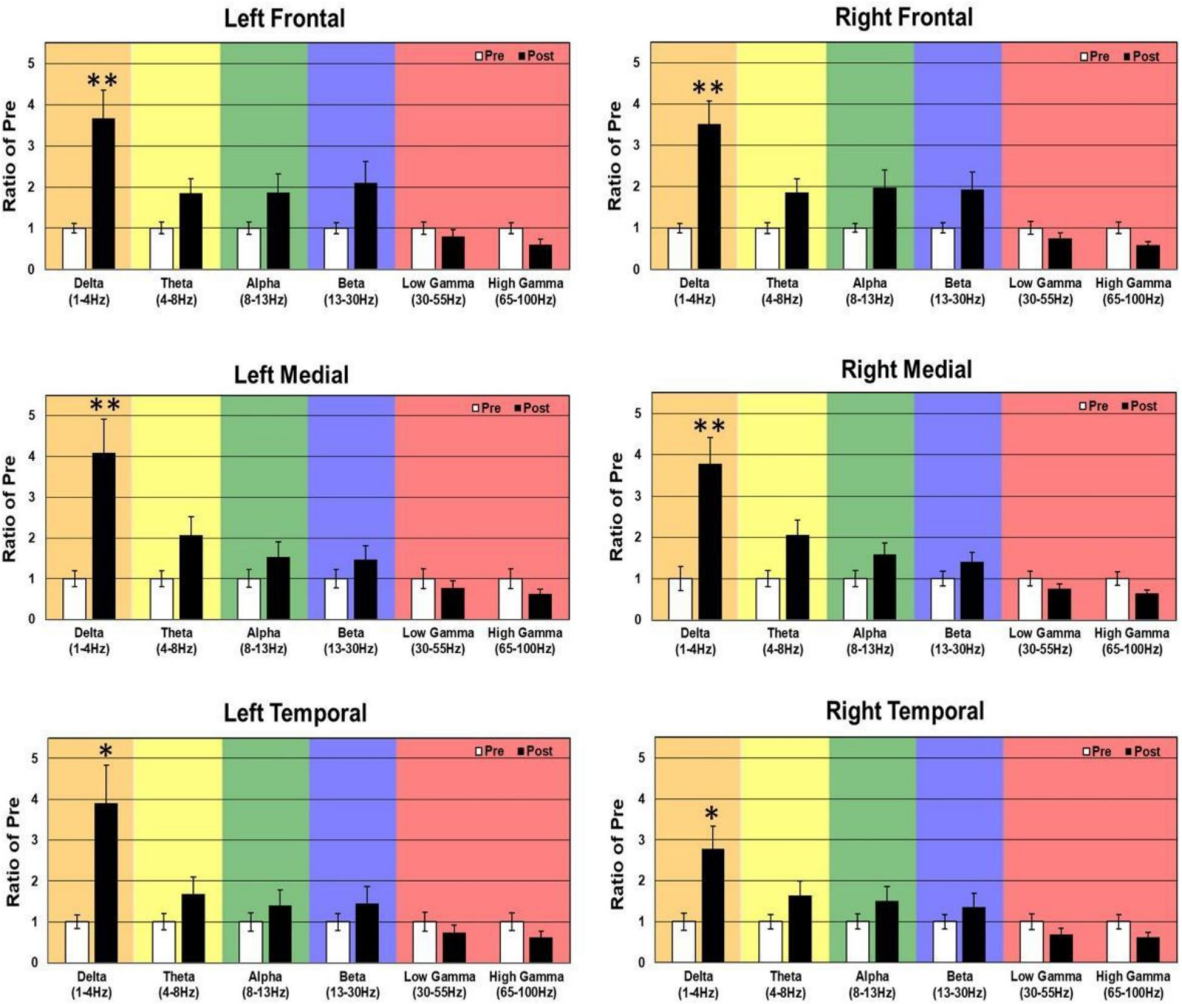
Effect of 2.5 mg/kg RBAC on EEG in *Fmr1* KO mice. Ratio of KO post-2.5 mg/kg RBACAC to Pre EEG power across frequency bands for distinct cortical regions following treatment

**Fig. 5:**
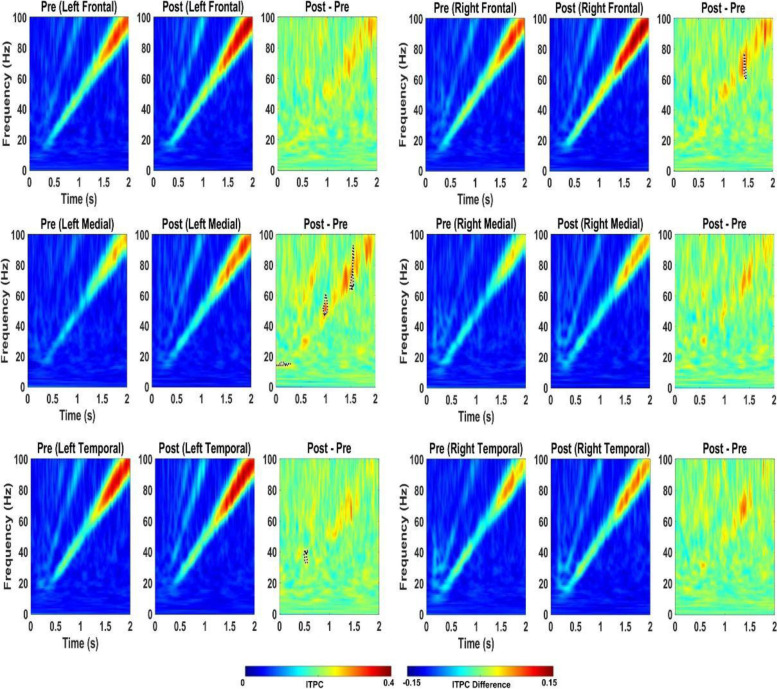
Effect of 2.5 mg/kg RBAC on auditory chirp in *Fmr1* KO mice. For each cortical region, the left panel shows the averaged inter-trial phase coherence (ITPC or phase locking factor) before vehicle treatment (Pre), the middle panel shows the averaged ITPC after vehicle treatment (Post) and the right panel shows Post–Pre. Significant increases in ITPC in Post compared to Pre are shown in black-outlined areas. Red areas in the right panels (Post–Pre) represent positive ITPC differences and blue areas represent negative ITPC differences

In WT mice, acute 2.5 mg/kg RBAC increased resting EEG power in delta, theta, alpha, and beta frequency bands (Fig. 2). Specifically, significant increases were observed in 5/6 brain regions for delta power, 2/6 for theta, 2/6 for alpha and 1/6 for beta (Fig. 2). With regard to auditory chirp studies, acute 2.5 mg/kg RBAC had no significant effect on chirp ITPC in WT mice (Fig. 3). In *Fmr1* KO mice, acute 2.5 mg/kg RBAC markedly increased EEG power in the delta frequency band (Fig. 4) throughout all brain areas. Thus, in comparison with WT mice, which demonstrated changes acutely across multiple frequency bands (Fig. 2), the acute effects of 2.5 mg/kg RBAC in *Fmr1* KO mice were more limited to increasing delta frequency power. Acute 2.5 mg/kg RBAC had slight but significant effects on increasing chirp ITPC in *Fmr1* KO mice (Fig. 5).

#### Effects of 5 mg/kg racemic baclofen on EEG in WT and KO mice

**Fig. 6:**
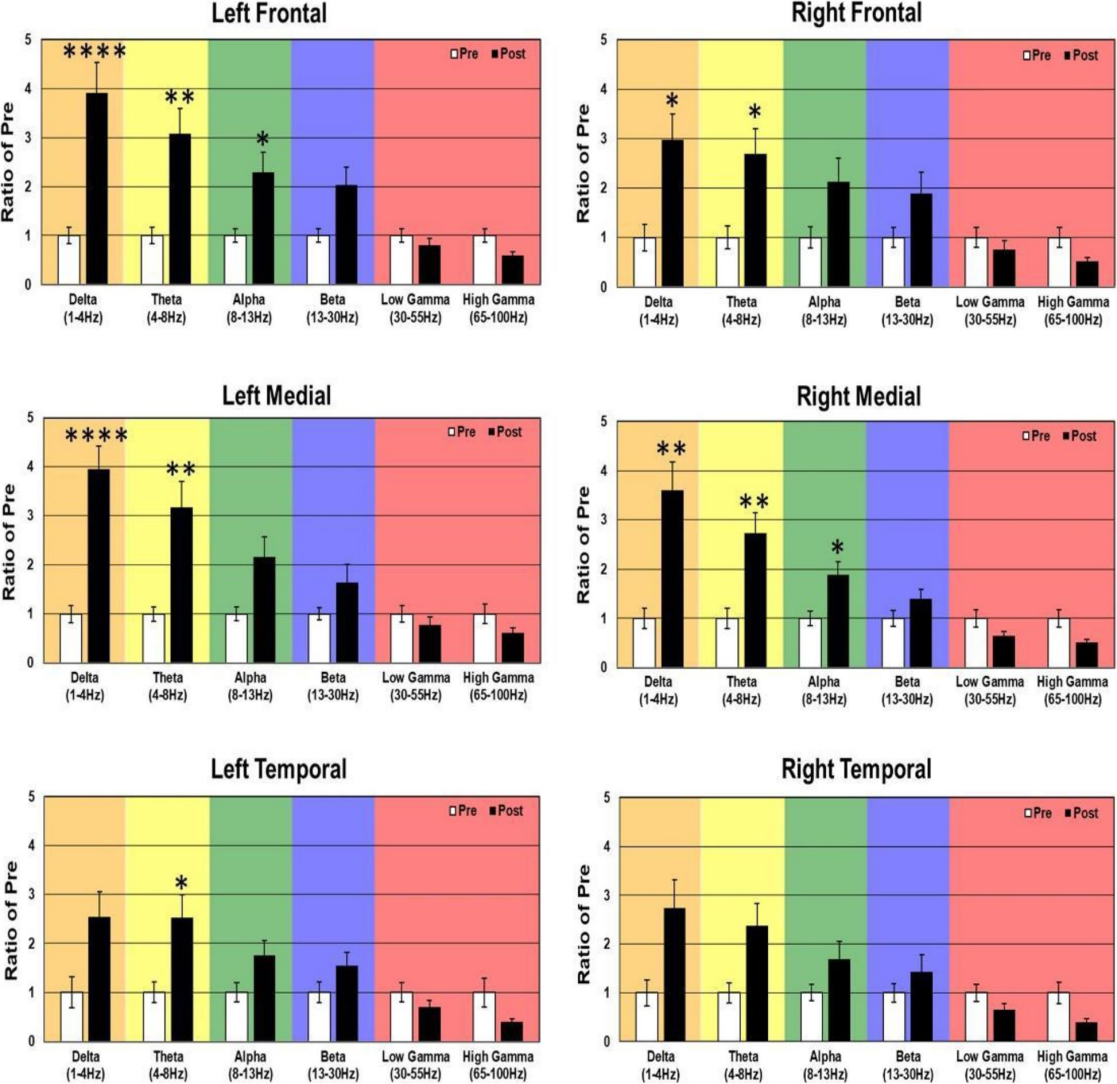
Effect of 5 mg/kg RBAC on EEG in WT mice. Ratio of WT post 5 mg/kg RBAC to Pre EEG power across frequency bands for distinct cortical regions following treatment

**Fig. 7:**
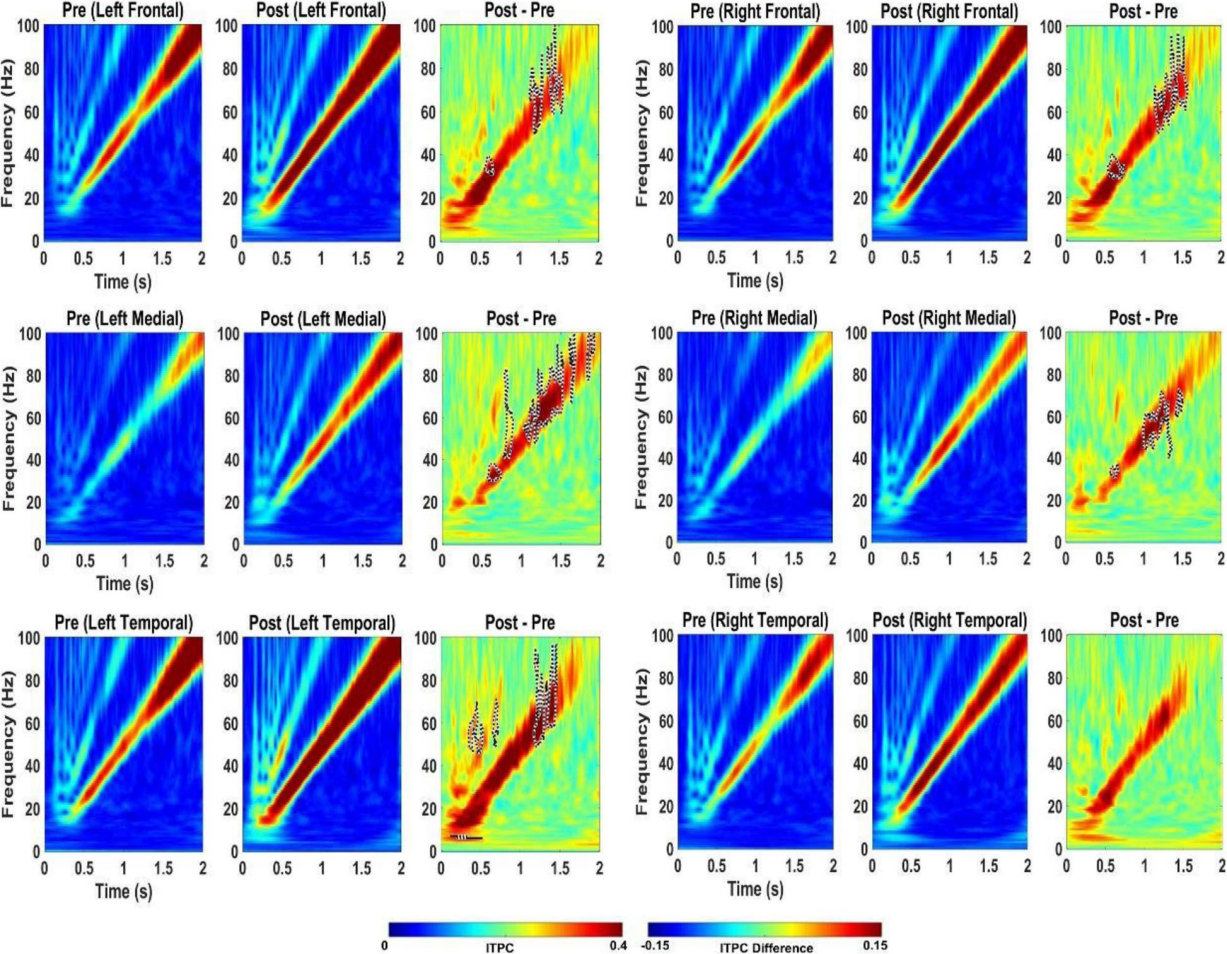
Effect of 5 mg/kg RBAC on auditory chirp in WT mice. For each cortical region, the left panel shows the averaged inter-trial phase coherence (ITPC or phase locking factor) before vehicle treatment (Pre), the middle panel shows the averaged ITPC after vehicle treatment (Post) and the right panel shows Post–Pre. Significant increases in ITPC in Post compared to Pre are shown in black-outlined areas. Red areas in the right panels (Post–Pre) represent positive ITPC differences and blue areas represent negative ITPC differences

**Fig. 8:**
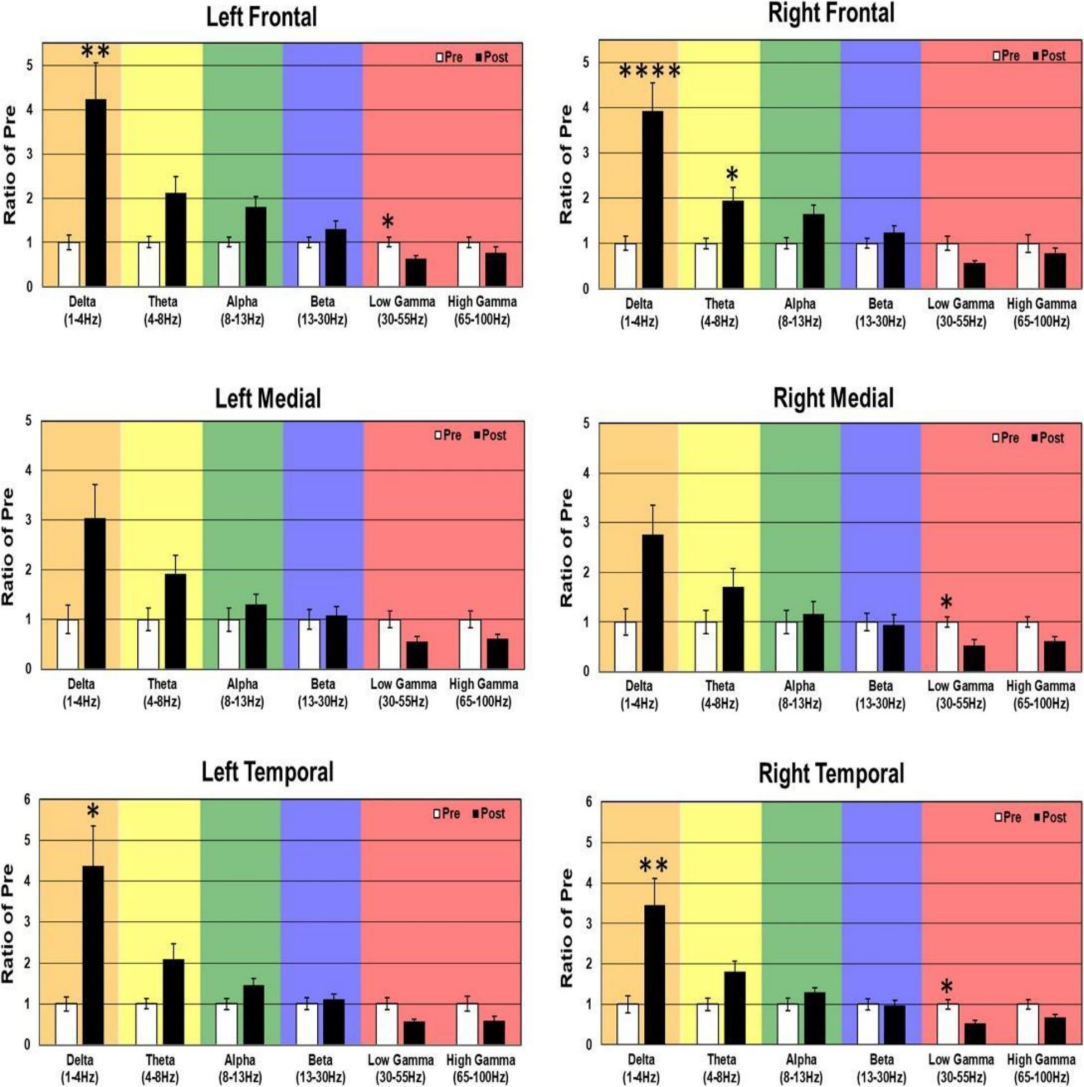
Effect of 5 mg/kg RBAC on EEG in *Fmr1* KO mice. Ratio of KO post 5 mg/kg RBAC to Pre EEG power across frequency bands for distinct cortical regions following treatment

**Fig. 9:**
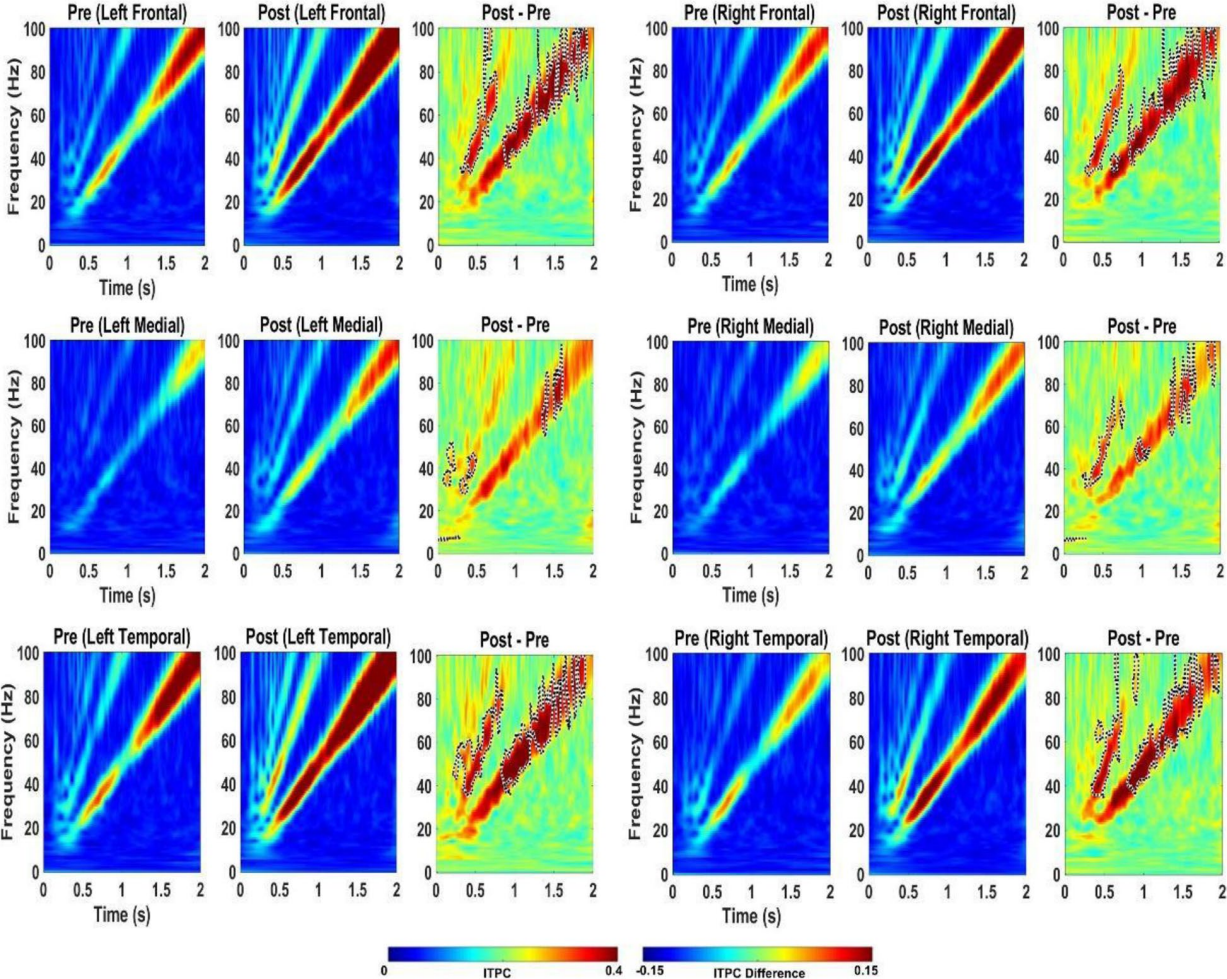
Effect of 5 mg/kg RBAC on auditory chirp in *Fmr1* KO mice. For each cortical region, the left panel shows the averaged inter-trial phase coherence (ITPC or phase locking factor) before vehicle treatment (Pre), the middle panel shows the averaged ITPC after vehicle treatment (Post) and the right panel shows Post–Pre. Significant increases in ITPC in Post compared to Pre are shown in black-outlined areas. Red areas in the right panels (Post–Pre) represent positive ITPC differences and blue areas represent negative ITPC differences

In WT mice, acute 5 mg/kg RBAC increased resting EEG power in delta, theta, and alpha frequency bands (Fig. 6). Specifically, significant increases were observed in 4/6 brain regions for delta power, 5/6 for theta, and 2/6 for alpha (Fig. 6). These changes occurred globally across the brain but the magnitude of the effects was region-specific. Acute 5 mg/kg RBAC markedly increased chirp ITPC in WT mice throughout the brain (Fig. 7). In *Fmr1* KO mice, acute 5 mg/kg RBAC increased EEG power in delta and theta frequency bands (Fig. 8). Specifically, significant increases were observed in 4/6 regions for delta power and 1/6 for theta power (Fig. 8). In addition, low gamma EEG power was suppressed in 3/6 areas (left frontal, right medial and right temporal areas (Fig. 8). Acute 5 mg/kg RBAC markedly increased chirp ITPC in *Fmr1* KO mice throughout the brain (Fig. 9).

#### EEG response to 30 mg acute RBAC treatment in humans with FXS

**Fig. 10:**
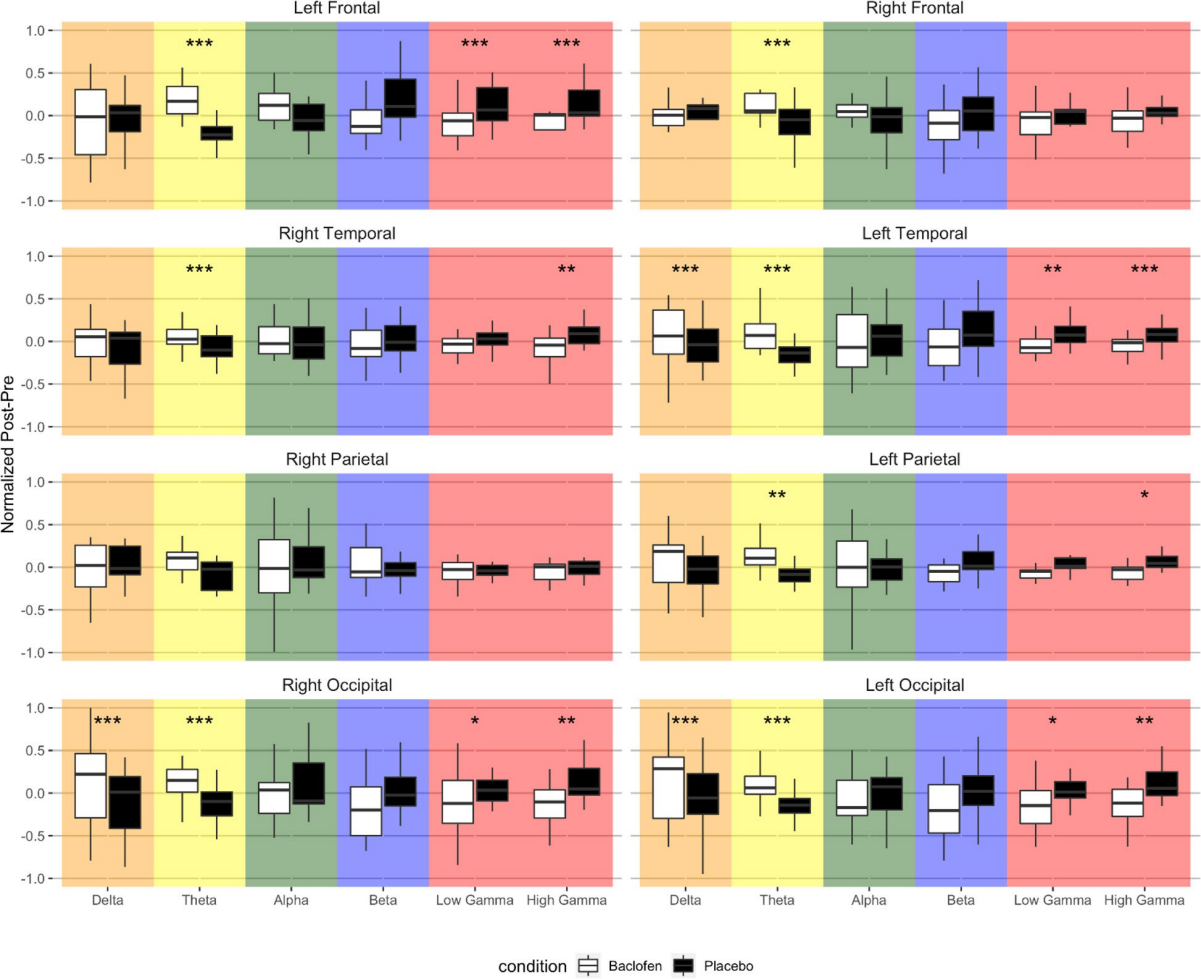
Effect of acute single-dose RBAC on resting-state EEG in FXS participants (*n* = 17). Pairs of boxplots visualizing standardized changes in relative power associated with either baclofen (white bars) or placebo (black bars) acute dose challenge across cortical region and grouped by frequency band. Significant differences (least-squared means contrasts) between baclofen and placebo treatment effects are designated with asterisks above each boxplot pair and corrected by a 5% false discovery rate (FDR). Cortical regions: L, left; R, right; F, frontal; T, temporal; P, parietal; O, occipital. Significance of adjusted *p* values: *, *p* < .05; **, *p* < .01, ***, *p* < .001

**Fig. 11:**
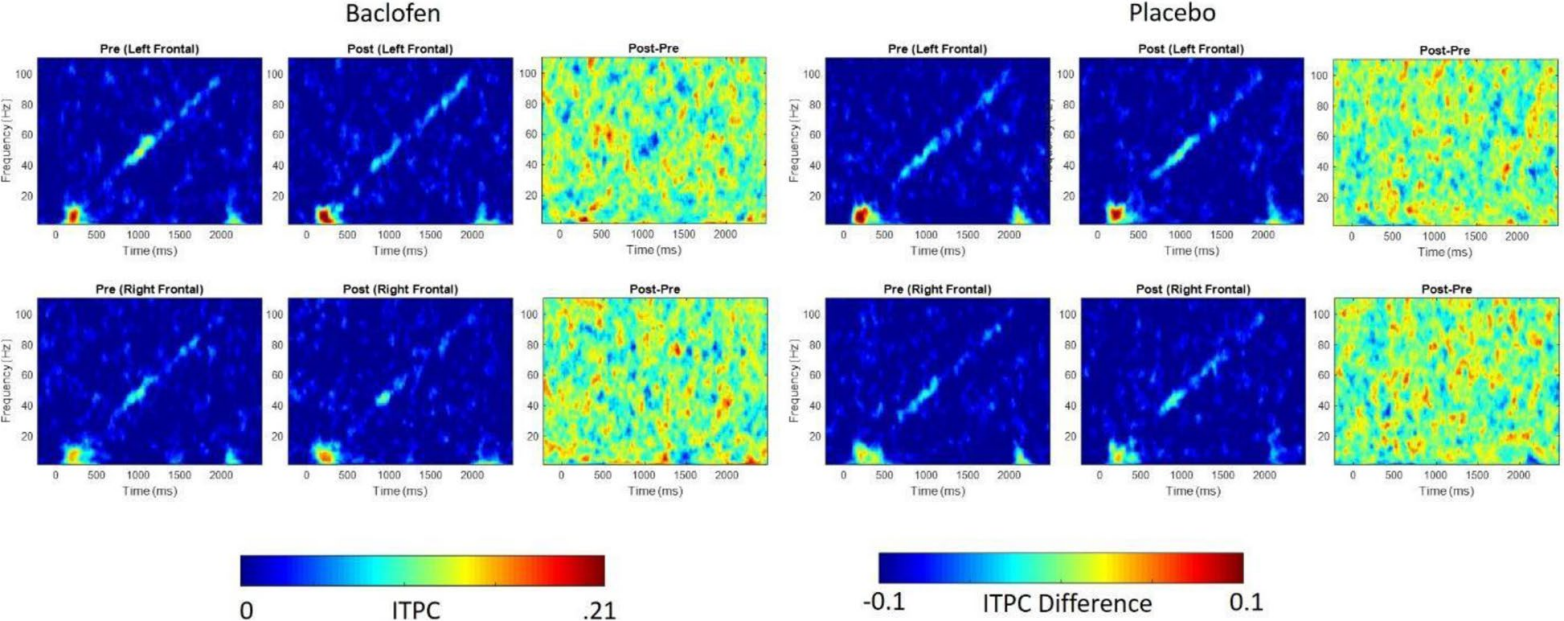
Effect of acute single-dose RBAC on auditory chirp in FXS participants. For left (upper row) and right (lower row) hemispheres, and for baclofen (left 3 columns) and placebo (right 3 columns) the left panel shows the averaged inter-trial phase coherence (ITPC or phase locking factor) before treatment (Pre), the middle panel shows the averaged ITPC after treatment (Post) and the right panel shows Post–Pre. Red areas in the right panels (Post–Pre) represent positive ITPC differences and blue areas represent negative ITPC differences

Human EEG resting state LMM analysis (*n* = 17) revealed a significant interaction effect between condition (RBAC vs. placebo), frequency band, and cortical region (*F*_42,7012_ = 2.4, *p* < .0001) on change in power (post-dose–pre-dose). Significant least-squared mean contrasts (5% FDR corrected) are presented in Fig. 10. Notably, significant RBAC-associated increases in theta power and reductions in gamma power were noted across the left frontal, bilateral temporal, and bilateral occipital regions in our human analysis. In the 11 subject sub-sample with evaluable chirp data across pre- and post-drug and placebo treatment, no significant RBAC-associated effects were noted in the human chirp analysis (Fig. 11).

## Discussion

In this paper, that is focused on FXS, we used 30-channel mouse skull surface MEA [22, 23] and 128-channel human EEG to test the acute effects of the GABA_B_ agonist RBAC on translational EEG biomarkers across species. In the mouse studies, we tested RBAC at 2 doses (2.5 mg/kg and 5 mg/kg and saline in parallel groups) in two genotypes (WT and *Fmr1* KO mice) and humans with FXS received a single 30 mg RBAC dose (approximately equivalent to mouse 5 mg/kg) or placebo in random order with a washout period between dosing. First, we found that 2.5 mg/kg and 5 mg/kg RBAC increase low-frequency band EEG resting power in WT mice. Additionally, 2.5 mg/kg (Fig. 4) and 5 mg/kg (Fig. 8) RBAC increases delta band EEG power in *Fmr1* KO mice. With the chirp stimuli, 5 mg/kg (Fig. 9), but not 2.5 mg/kg (Fig. 5), RBAC significantly increases chirp ITPC (phase synchronization) in *Fmr1* KO mice. In our small human subject sub-sample, 30 mg acute RBAC dosing did not significantly increase chirp ITPC, but consistent with our mouse data, did significantly increase theta power and reduce gamma power at rest.

Our across species resting state power findings show similar effects of RBAC on EEG biomarkers. In the mice, 5 mg/kg of RBAC increased delta band power diffusely and reduced low gamma power in left frontal, right medial and right temporal brain regions. In humans, 30 mg acute RBAC dosing was associated with theta band power increase and gamma band power reduction in left frontal and bilateral temporal and occipital regions compared to placebo. These findings are important in demonstrating proof of principle that EEG biomarkers of drug effects show translational synchrony across species in FXS. Additionally, this work shows the ability of a single small molecule drug dose to potentially normalize aberrant resting neurooscillatory activity in FXS across species.

There is increasing distinction among the role of gamma oscillations such a general association with brain activation as well as increases in precise synchrony of cognitive processes, gamma oscillations hold a special interest in neurodevelopmental conditions because of their relation to cortical excitability [46, 47], association with cognitive processes [48], and analogous measurability in animal models [22]. The role of gamma oscillations is increasingly nuanced, such that precise synchrony in gamma activity is contributory to higher-order cognition [49, 50], and that a modest degree of asynchrony or noise represents physiological processes [51–53]. Nevertheless, asynchronous (usually broadband) gamma power, above what is typically expected, has been associated with disease states [48] as well as with reduced spike precision and spectral leakage of spiking activities in microcircuit preparations [54].

At baseline, gamma power has shown to be consistently increased at rest in humans with FXS, and here we demonstrated acute RBAC treatment significantly reduced this abnormality. Though multiple physiological roles have been identified for gamma oscillations [51–53], FXS patients precise gamma synchrony to sensory input is impaired [18] and asynchronous background gamma activity is increased [18, 25, 55]. In full mutation, non-mosaic males with FXS, increased gamma power is associated with increased severity across behavioral domains (i.e., irritability, abnormal speech, hyperactivity) as well as cognitive function [55]. Our finding of co-occurring RBAC-associated theta band power increase and gamma band reduction is consistent with our prior hypothesis that in FXS increased theta may be recruited to downregulate gamma activity and thus potentially suppress sensory hyperexcitability [25] thus showing a potential mechanistic signature of RBAC use in FXS. Regarding mechanisms of gamma power modulation by GABAergic mechanisms, several lines of evidence have identified GABAergic interneurons as the primary source of gamma oscillations in the brain [56–59]. To our knowledge, only one previous study [36] in mice reported gamma power reduction following acute administration of racemic baclofen. Our data concur with this finding that GABA_B_ modulation in particular may suppress resting gamma power in the brain.

Another remarkable similarity in the effects of RBAC on both mouse and human EEG in our study is increases in low-frequency power. In the mouse, this manifests as diffuse increases in delta power and also a trend toward increases in theta power in all regions. This varied slightly by dose and genotype (cf. Figs. 2, 4, 6, and 8), but overall increases in low-frequency power were consistently observed. In the human EEG, single-dose RBAC significantly increased delta power in 3/8 regions and theta power in 7/8 regions (Fig. 10). Increases in low-frequency power were previously observed ([36], Fig. 2B). Our view based on the data we have generated and that from [36] is that GABA_B_ agonism by RBAC clearly “slows” the EEG, meaning increasing power in the low frequency bands. This has been observed in multiple other contexts with GABA agonism causing synchronization and generation of “slow waves” in the delta frequency band [60, 61]. In particular, a quantitative EEG study with GABA_B_ agonists including RBAC has shown increases in delta frequency power when administered acutely to mice [60]. We noted that the higher dose of RBAC (5 mg/kg) given acutely to mice had sedative effects, which was also observed by [36, 60].

A strength of our approach with RBAC target engagement study across species is a similar approach to resting state electrophysiology analysis and the demonstration of specific parallel drug effects across species. However, our human sample was small, and included a mixture of males and females with varying clinical levels of function. Future work will be required with larger human sample sizes to determine if there is a subset of persons with FXS who are RBAC treatment responders. It is also possible that subgroups of the FXS population have electrophysiological RBAC target engagement while others may not, which might underlie treatment outcome variability. This is of critical importance given that while FXS is a single gene disorder, the clinical presentations of the disorder vary widely. Additionally, we have demonstrated that the EEG signatures in FXS differ based on sex [62], and therefore it is possible that EEG target engagement also may differ based upon the sex of the subject. Thus, increasing our human sample size in this line of research will be essential to answer these questions of importance to the field.

Chirp analyses in the human data were underpowered due to data loss and reduced trial count relative to the murine task. Collecting more trials to ensure adequate sampling may be needed for future studies. It is also possible that single dose RBAC is not sufficient in human participants to achieve the effects on chirp phase dynamics. In the future, evaluating evoked EEG responses following baclofen treatment may further advance mechanistic understanding, provide additional translational electrophysiology tools for testing drugs like RBAC, and directly evaluate the relation of functional brain alterations to disturbances in sensory and cognitive function. Our human work also will benefit from dose finding study to determine a minimal effective dose that positively engages the human neurophysiology of FXS while also potentially improving performance-based clinical measures. Human and mouse future investigation of chronic RBAC use also will be essential to understanding the true potential clinical utility of this compound in FXS and its effect on brain function in the context of longer-term treatment.

## Conclusion

Improving translational synchrony of outcome measures across animal and human study is an essential element to success of drug development in the FXS and other neurodevelopmental disorder fields. In this study, we report the feasibility and successful effort to establish parallel target engagement across species using EEG biomarkers. We demonstrated an analogous change in resting low-frequency power (increase) and gamma band (reduction) activity in mouse and human study, two alterations we have previously related to clinical features of the illness [6]. More work is required to clarify dose-response patterns in beneficial and potentially adverse effects, and to identify potential baseline EEG characteristics, molecular aspects of FXS, sex, age, and other features that predict RBAC response, as such findings would help individualize patient care and potentially stratify patients in clinical trials. Synchronized preclinical and human study is a model that can de-risk large-scale trials and drug development programs in this field to guide more optimal and informed “go no-go” decisions during treatment development efforts.

## Supplementary Information

**Additional file 1: Supplemental Figure S1**. Pre-treatment resting EEG comparison of FMR1 KO and WT mice.

**Additional file 2: Supplemental Figure S2**. Pre-treatment EEG auditory chirp comparison of FMR1 KO and WT mice.

## Data Availability

The datasets used and/or analyzed during the current study are available from the corresponding author on reasonable request.

## Abbreviations

ASD: Autism spectrum disorders
EEG: Electroencephalography
FMRP: Fragile X mental retardation protein
FXP: Fragile X protein
FXS: Fragile X syndrome
ITPC: inter-trial phase coherence (phase locking factor)
K/X: Ketamine/xylazine
MEA: Multielectrode array
RBAC: Racemic baclofen

## References

[1] Crawford DC, Acuña JM, Sherman SL (2001). FMR1 and the fragile X syndrome: human genome epidemiology review. Genetics Med.

[2] Yu S, Pritchard M, Kremer E, Lynch M, Nancarrow J, Baker E (1991). Fragile X genotype characterized by an unstable region of DNA. Science..

[3] Darnell Jennifer C, Van Driesche SJ, Zhang C, Hung Ka Ying S, Mele A, Fraser Claire E (2011). FMRP stalls ribosomal translocation on mrnas linked to synaptic function and autism. Cell..

[4] Abbeduto L, Hagerman RJ (1997). Language and communication in fragile X syndrome. Ment Retard Dev Disabil Res Rev.

[5] Berry-Kravis E (2002). Epilepsy in fragile X syndrome. Dev Med Child Neurol.

[6] Hagerman RJ, Berry-Kravis E, Kaufmann WE, Ono MY, Tartaglia N, Lachiewicz A (2009). Advances in the treatment of fragile X syndrome. Pediatrics..

[7] Miller LJ, McIntosh DN, McGrath J, Shyu V, Lampe M, Taylor AK (1999). Electrodermal responses to sensory stimuli in individuals with fragile X syndrome: a preliminary report. Am J Med Genet.

[8] Musumeci SA, Hagerman RJ, Ferri R, Bosco P, Bernardina BD, Tassinari CA (1999). Epilepsy and EEG findings in males with fragile X syndrome. Epilepsia..

[9] Roberts JE, Hatton DD, Bailey DB (2001). Development and behavior of male toddlers with fragile X syndrome. J Early Interv.

[10] Sabaratnam M, Vroegop PG, Gangadharan SK (2001). Epilepsy and EEG findings in 18 males with fragile X syndrome. Seizure..

[11] Sinclair D, Oranje B, Razak KA, Siegel SJ, Schmid S (2017). Sensory processing in autism spectrum disorders and Fragile X syndrome-From the clinic to animal models. Neurosci Biobehav Rev.

[12] Van der Molen MJW, Huizinga M, Huizenga HM, Ridderinkhof KR, Van der Molen MW, Hamel BJC (2010). Profiling Fragile X Syndrome in males: strengths and weaknesses in cognitive abilities. Res Dev Disabil.

[13] Wisniewski KE, Segan SM, Miezejeski CM, Sersen EA, Rudelli RD (1991). The fra(X) syndrome: Neurological, electrophysiological, and neuropathological abnormalities. Am J Med Genet.

[14] Schmitt LM, Wang J, Pedapati EV, Thurman AJ, Abbeduto L, Erickson CA (2020). A neurophysiological model of speech production deficits in fragile X syndrome. Brain. Communications..

[15] Erickson CA, Kaufmann WE, Budimirovic DB, Lachiewicz A, Haas-Givler B, Miller RM, et al. Best Practices in Fragile X Syndrome Treatment Development. Brain Sci. 2018;8(12). 10.3390/brainsci8120224 PMC6315698 30558274

[16] Castrén M, Paakkonen A, Tarkka IM, Ryynanen M, Partanen J (2003). Augmentation of auditory N1 in children with fragile X syndrome. Brain Topogr.

[17] Schneider A, Leigh MJ, Adams P, Nanakul R, Chechi T, Olichney J (2013). Electrocortical changes associated with minocycline treatment in fragile X syndrome. J Psychopharmacol.

[18] Ethridge LE, De Stefano LA, Schmitt LM, Woodruff NE, Brown KL, Tran M (2019). Auditory EEG Biomarkers in Fragile X Syndrome: Clinical Relevance. Front Integr Neurosci.

[19] Berry-Kravis EM, Lindemann L, Jønch AE, Apostol G, Bear MF, Carpenter RL (2018). Drug development for neurodevelopmental disorders: lessons learned from fragile X syndrome. Nat Rev Drug Discov.

[20] Ewen JB, Sweeney JA, Potter WZ (2019). Conceptual, regulatory and strategic imperatives in the early days of EEG-based biomarker validation for neurodevelopmental disabilities. Front Integr Neurosci.

[21] Sahin M, Jones SR, Sweeney JA, Berry-Kravis E, Connors BW, Ewen JB, et al. Discovering translational biomarkers in neurodevelopmental disorders. Nat Rev Drug Discov. 2018. 10.1038/d41573-018-00010-7 PMC7556736 30936503

[22] Jonak CR, Lovelace JW, Ethell IM, Razak KA, Binder DK (2018). Reusable multielectrode array technique for electroencephalography in awake freely moving mice. Front Integr Neurosci.

[23] Jonak CR, Lovelace JW, Ethell IM, Razak KA, Binder DK (2020). Multielectrode array analysis of EEG biomarkers in a mouse model of Fragile X Syndrome. Neurobiol Dis.

[24] Ethridge LE, White SP, Mosconi MW, Wang J, Pedapati EV, Erickson CA (2017). Neural synchronization deficits linked to cortical hyper-excitability and auditory hypersensitivity in fragile X syndrome. Mol Autism.

[25] Wang J, Ethridge LE, Mosconi MW, White SP, Binder DK, Pedapati EV (2017). A resting EEG study of neocortical hyperexcitability and altered functional connectivity in fragile X syndrome. J Neurodev Disord.

[26] Ethridge LE, White SP, Mosconi MW, Wang J, Byerly MJ, Sweeney JA (2016). Reduced habituation of auditory evoked potentials indicate cortical hyper-excitability in Fragile X Syndrome. Transl Psychiatry.

[27] Wen TH, Lovelace JW, Ethell IM, Binder DK, Razak KA (2019). Developmental Changes in EEG Phenotypes in a Mouse Model of Fragile X Syndrome. Neuroscience..

[28] Rais M, Binder DK, Razak KA, Ethell IM (2018). Sensory Processing Phenotypes in Fragile X Syndrome. ASN Neuro.

[29] Lovelace JW, Ethell IM, Binder DK, Razak KA (2018). Translation-relevant EEG phenotypes in a mouse model of Fragile X Syndrome. Neurobiol Dis.

[30] Gantois I, Vandesompele J, Speleman F, Reyniers E, D'Hooge R, Severijnen LA (2006). Expression profiling suggests underexpression of the GABA(A) receptor subunit delta in the fragile X knockout mouse model. Neurobiol Dis.

[31] Heulens I, D'Hulst C, Van Dam D, De Deyn PP, Kooy RF (2012). Pharmacological treatment of fragile X syndrome with GABAergic drugs in a knockout mouse model. Behav Brain Res.

[32] Pacey LK, Heximer SP, Hampson DR (2009). Increased GABA(B) receptor-mediated signaling reduces the susceptibility of fragile X knockout mice to audiogenic seizures. Mol Pharmacol.

[33] Berry-Kravis EM, Hessl D, Rathmell B, Zarevics P, Cherubini M, Walton-Bowen K (2012). Effects of STX209 (Arbaclofen) on neurobehavioral function in children and adults with fragile X syndrome: a randomized, controlled, Phase 2 Trial. Sci Transl Med.

[34] Henderson C, Wijetunge L, Kinoshita MN, Shumway M, Hammond RS, Postma FR (2012). Reversal of disease-related pathologies in the fragile X mouse model by selective activation of GABAB receptors with arbaclofen. Sci Transl Med.

[35] Kang JY, Chadchankar J, Vien TN, Mighdoll MI, Hyde TM, Mather RJ (2017). Deficits in the activity of presynaptic gamma-aminobutyric acid type B receptors contribute to altered neuronal excitability in fragile X syndrome. J Biol Chem.

[36] Sinclair D, Featherstone R, Naschek M, Nam J, Du A, Wright S, et al. GABA-B agonist baclofen normalizes auditory-evoked neural oscillations and behavioral deficits in the Fmr1 knockout mouse model of fragile X syndrome. Eneuro. 2017;4 ENEURO.0380-16.2017. 10.1523/ENEURO.0380-16.2017 PMC5394929 28451631

[37] Bakker CE, al. e, Verheij C, Willemsen R, Helm Rvd, Oerlemans F, et al. Fmr1 knockout mice: a model to study fragile X mental retardation. The Dutch-Belgian Fragile X Consortium. Cell. 1994;78(1):23-33. 8033209

[38] Nair AB, Jacob S (2016). A simple practice guide for dose conversion between animals and human. J Basic Clin Pharm.

[39] Jonak CR, Sandhu MS, Assad SA, Barbosa JA, Makhija M, Binder DK (2021). Correction to: the PDE10A inhibitor TAK-063 reverses sound-evoked EEG abnormalities in a mouse model of fragile X syndrome. Neurotherapeutics..

[40] Jonak CR, Sandhu MS, Assad SA, Barbosa JA, Makhija M, Binder DK (2021). The PDE10A Inhibitor TAK-063 reverses sound-evoked EEG abnormalities in a mouse model of fragile X syndrome. Neurotherapeutics..

[41] Desikan RS (2006). An automated labeling system for subdividing the human cerebral cortex on MRI scans into gyral based regions of interest. NeuroImage.

[42] Artieda J, Valencia M, Alegre M, Olaziregi O, Urrestarazu E, Iriarte J (2004). Potentials evoked by chirp-modulated tones: a new technique to evaluate oscillatory activity in the auditory pathway. Clin Neurophysiol.

[43] Pérez-Alcázar M, Nicolás MJJ, Valencia M, Alegre M, Iriarte J, Artieda J (2008). Chirp-evoked potentials in the awake and anesthetized rat. A procedure to assess changes in cortical oscillatory activity. Exp Neurol.

[44] Purcell DW, John SM, Schneider BA, Picton TW (2004). Human temporal auditory acuity as assessed by envelope following responses. J Acoustical Soc Am.

[45] Tallon-Baudry C, Bertrand O, Delpuech C, Pernier J (1996). Stimulus specificity of phase-locked and non-phase-locked 40 Hz visual responses in human. J Neurosci.

[46] Goswami S, Cavalier S, Sridhar V, Huber KM, Gibson JR (2019). Local cortical circuit correlates of altered EEG in the mouse model of Fragile X syndrome. Neurobiol Dis.

[47] Antoine MW, Langberg T, Schnepel P, Feldman DE (2019). Increased Excitation-Inhibition Ratio Stabilizes Synapse and Circuit Excitability in Four Autism Mouse Models. Neuron.

[48] Mably AJ, Colgin LL (2018). Gamma oscillations in cognitive disorders. Curr Opin Neurobiol.

[49] Fries P (2015). Rhythms for Cognition: Communication through Coherence. Neuron.

[50] Fries P (2005). A mechanism for cognitive dynamics: neuronal communication through neuronal coherence. Trends Cogn Sci.

[51] Burke JF (2013). Synchronous and asynchronous theta and gamma activity during episodic memory formation. J Neurosci.

[52] Brunel N, Hansel D (2006). How noise affects the synchronization properties of recurrent networks of inhibitory neurons. Neural Comput.

[53] Battaglia D, Hansel D (2011). Synchronous chaos and broad band gamma rhythm in a minimal multi-layer model of primary visual cortex. PLoS Comput Biol.

[54] Guyon N (2021). Network Asynchrony Underlying Increased Broadband Gamma Power. J Neurosci.

[55] Pedapati EV, Schmitt LM, Ethridge LE, Miyakoshi M, Sweeney JA, Liu R, et al. Neocortical localization and thalamocortical modulation of neuronal hyperexcitability contribute to Fragile X Syndrome. Nat Commun Biol 2022.25 10.1038/s42003-022-03395-9 PMC9095835 35546357

[56] Traub RD, Whittington MA, Colling SB, Buzsaki G, Jefferys JG (1996). Analysis of gamma rhythms in the rat hippocampus in vitro and in vivo. J Physiol.

[57] Bartos M, Vida I, Jonas P (2007). Synaptic mechanisms of synchronized gamma oscillations in inhibitory interneuron networks. Nat Rev Neurosci.

[58] Sohal VS, Zhang F, Yizhar O, Deisseroth K (2009). Parvalbumin neurons and gamma rhythms enhance cortical circuit performance. Nature..

[59] McNally JM, Aguilar DD, Katsuki F, Radzik LK, Schiffino FL, Uygun DS (2021). Optogenetic manipulation of an ascending arousal system tunes cortical broadband gamma power and reveals functional deficits relevant to schizophrenia. Mol Psychiatry.

[60] Vienne J, Bettler B, Franken P, Tafti M (2010). Differential effects of GABAB receptor subtypes, {gamma}-hydroxybutyric Acid, and Baclofen on EEG activity and sleep regulation. J Neurosci.

[61] Vienne J, Lecciso G, Constantinescu I, Schwartz S, Franken P, Heinzer R (2012). Differential effects of sodium oxybate and baclofen on EEG, sleep, neurobehavioral performance, and memory. Sleep..

[62] Smith EG, Pedapati EV, Liu R, Schmitt LM, Dominick KC, Shaffer RC (2021). Sex differences in resting EEG power in Fragile X Syndrome. J Psychiatr Res.

