# Supplementary figures and images for: Baclofen-associated neurophysiologic target engagement across species in fragile X syndrome
Source: J Neurodev Disord. 2022 Sep 27;14:52. doi: 10.1186/s11689-022-09455-9 (PMC9513876; doi:10.1186/s11689-022-09455-9)

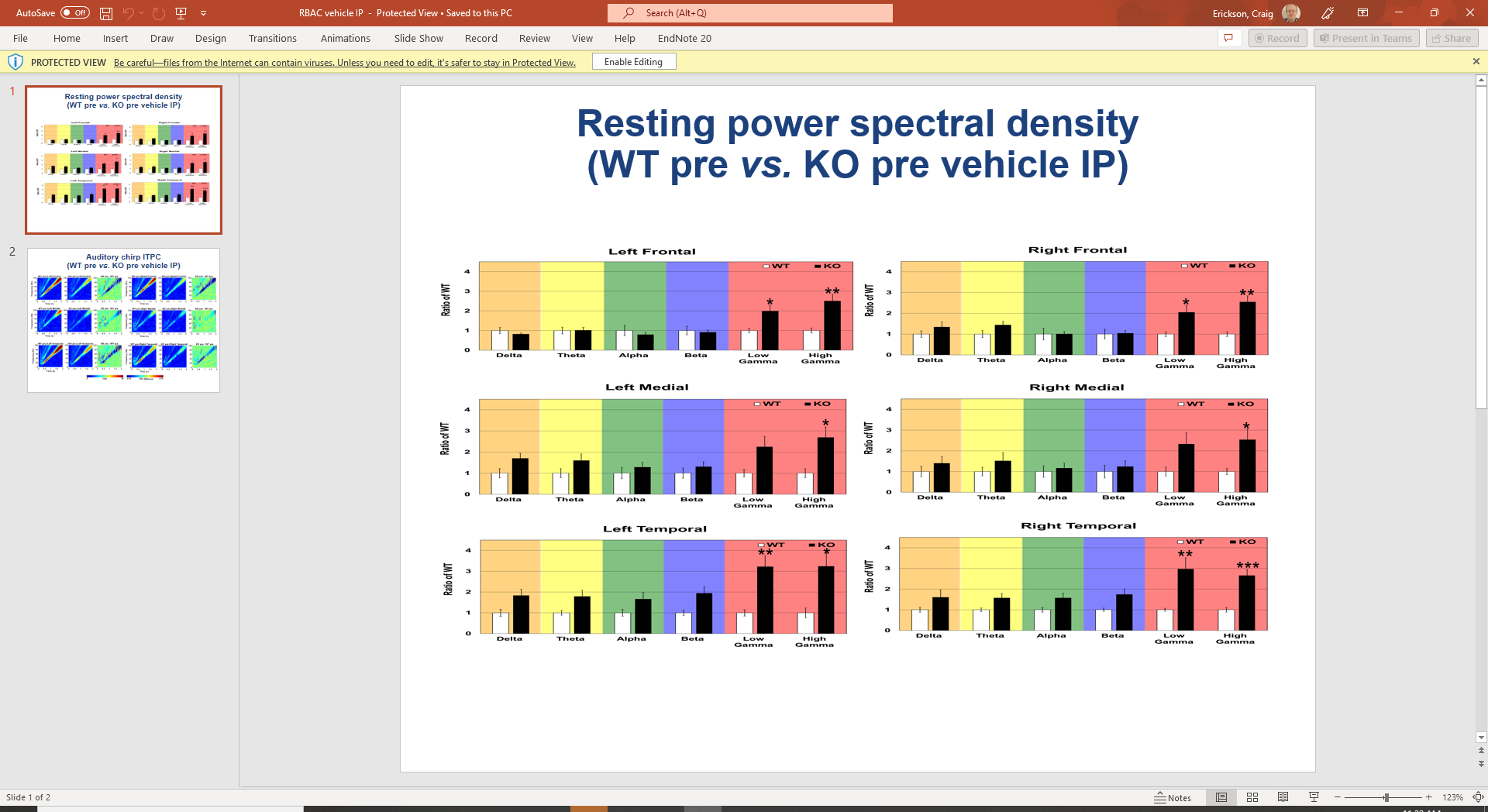

Supplement: Supplementary file 1 — Additional file 1: Supplemental Figure S1. Pre-treatment resting EEG comparison of FMR1 KO and WT mice. [file 11689_2022_9455_MOESM1_ESM.docx]

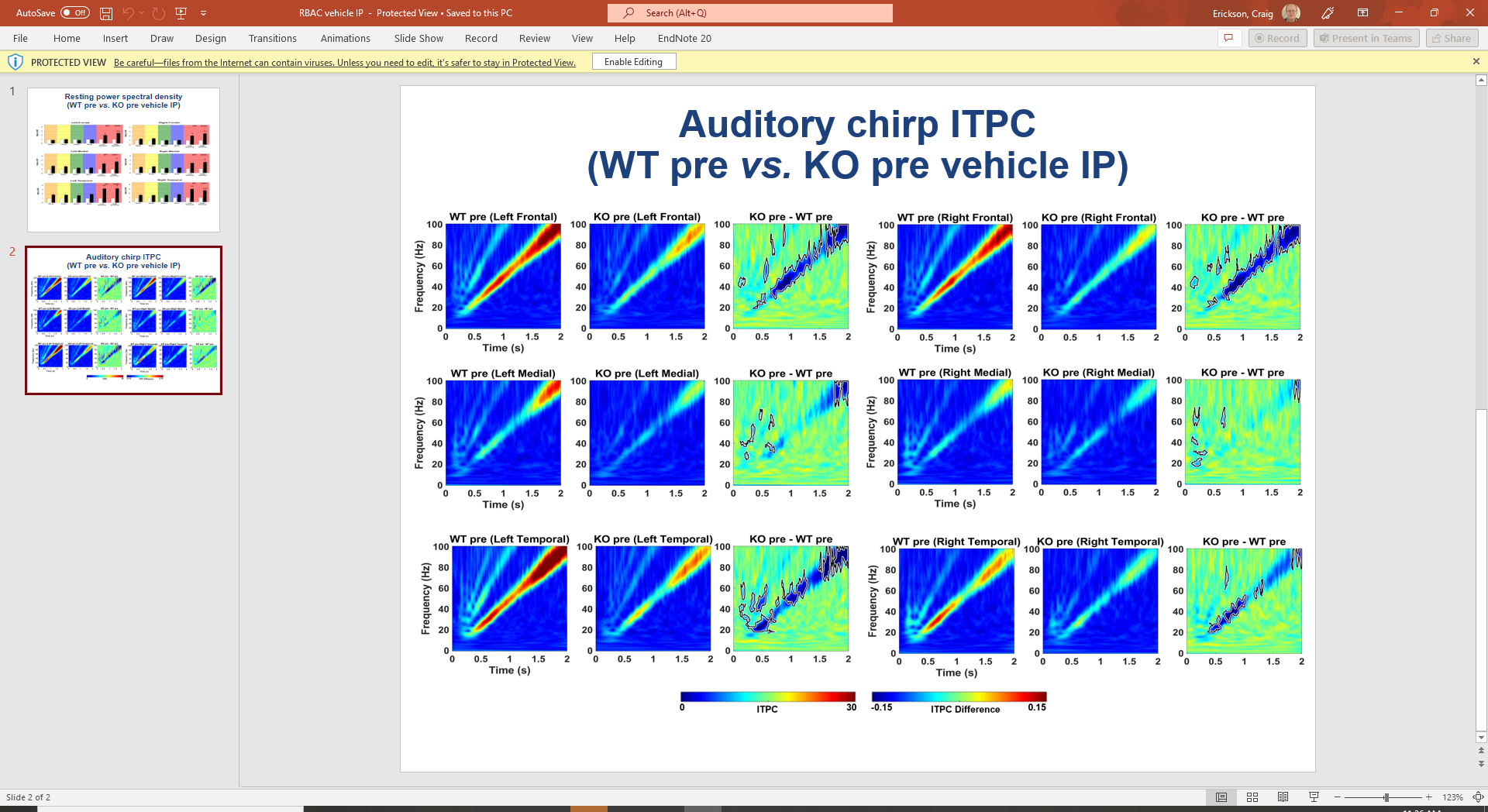

Supplement: Supplementary file 2 — Additional file 2: Supplemental Figure S2. Pre-treatment EEG auditory chirp comparison of FMR1 KO and WT mice. [file 11689_2022_9455_MOESM2_ESM.docx]
